# The seas approach reduced Cobb angle, and the thera-band exercise improved scapula winging in a double major curve type of Adolescent Idiopathic Scoliosis (AIS) – a case report

**DOI:** 10.1186/1748-7161-9-S1-P3

**Published:** 2014-12-04

**Authors:** Chiiko Ishihara, Yosuke Shiraishi

**Affiliations:** 1 Kojimachi Dr.Shiraishi Osteopath, Tokyo, Japan

## Background

In Japan, bracing is the only treatment which is considered as an effective conservative treatment for AIS. Therefore, therapeutic exercise for scoliosis does not pervade as a clinical approach for AIS. Only a few orthopaedists and therapists adopt Side Shift and Pelvic Hitch exercise. In general, clinical evidence regarding the effectiveness of specific exercise for preventing progression of curves is not recognized. In this case, Cobb angle progressed 9 degrees post bracing.

## Purpose

To report:

1. ASC (active self correction) of SEAS is effective in reducing Cobb angle of the patient after skeletal mature.

2. Thera-band Shiraishi exercise is effective in improving scapula winging.

3. We want to spread that specific exercise is effective to AIS.

## Methods

We followed a 14 year-old girl diagnosed in 2006 with AIS, 28 degrees in the thoracic and 28 degrees in the lumbar. Started Boston brace immediately, with everyday exercise including Thera-band exercise for scapula winging and AKA (arthrokinematic approach). She finished bracing, at the age of 18, Cobb angle remained 31 degrees. After 6months, her curve progressed again, then we offered a night brace, ASC (active self correction) of SEAS and Mitsui-thermal therapy.

## Conclusion & discussion

In this case, a Cobb angle increased from 28 degrees to 31 degrees in the thoracic vertebrae during 14 to 18 years old. However, after 6 month from bracing off, thoracic curve increased from 31 to 37 degrees. When she was 21 years old, SEAS approach was applied and 3 months later, Cobb angle decreased from 37 to 30 degrees. SEAS approach was effective to Cobb angle improvement. In addition, we think that the thera-band exercise makes cosmetic improvement on scapula winging, and Mistui-thermotherapy build-up strength of the spinal muscles.

## Consent

Written informed consent was obtained from the parents/legal guardian of the patient for publication of this Case report. A copy of the written consent is available for review by the Editor of this journal.

